# Targeted Albumin Administration in Cardiac Surgery: Refining Perioperative Fluid Strategy

**DOI:** 10.31083/RCM45733

**Published:** 2025-11-11

**Authors:** Bingyang Ji, Shujie Yan, Man Huang

**Affiliations:** ^1^Department of Cardiopulmonary Bypass, Fuwai Hospital, National Center for Cardiovascular Disease, Chinese Academy of Medical Sciences & Peking Union Medical College, National Clinical Research Center for Cardiovascular Diseases, 100037 Beijing, China; ^2^Department of General Intensive Care Unit, The Second Affiliated Hospital of Zhejiang University School of Medicine, 310009 Hangzhou, Zhejiang, China

Cardiac surgery poses specific challenges in fluid management. The role of albumin administration during cardiac surgery has long been controversial. Although it has theoretical advantages, it is expensive and there is limited evidence that it improves clinical outcomes in cardiac surgery patients. However, over the past five years, evidence has emerged that albumin may be beneficial as a targeted therapy in selected patients.

The physiological rationale for albumin infusion is closely related to the pathophysiology of cardiopulmonary bypass (CPB). CPB can induce a systemic inflammatory response, disrupt cellular endothelial function, increase capillary permeability, and result in hypoalubinemia due to hemodilution and the adsorption of plasma proteins such as albumin by the CPB circuit. These changes lower plasma oncotic pressure which causes fluid to leak from the intravascular space, resulting in tissue edema. Albumin is the main determinant of plasma oncotic pressure, and physically retains approximately 18 mL of water per gram in the intravascular space. In addition, it binds drugs, hormones, and toxins, neutralizes reactive oxygen species, and may limit glycocalyx degradation. These beneficial properties of albumin provide a physiological rationale for considering its perioperative use in cardiac surgery.

However, recent clinical evidence does not support routine albumin use in adult cardiac surgery with CPB. The Albumin in Cardiac Surgery (ALBICS) trial, a double-blinded randomized controlled trial (RCT) in 1386 on-pump cardiac surgery patients, showed that 4% albumin versus Ringer’s lactate for CPB priming and perioperative fluid, did not reduce major adverse clinical events at 90 days, but was associated with increased perioperative bleeding, higher transfusion requirements, and the need for more reoperations [1, 2]. A recent network meta-analysis confirmed the increased need for perioperative Red Blood Cells (RBC) transfusion with albumin priming compared to using crystalloid solutions [3]. There have also been concerns regarding decreased renal function following the routine use of albumin during cardiac surgery. The Albumin Infusion and Acute Kidney Injury following Cardiac Surgery (ALBICS-AKI) trial involving high-risk cardiac surgery patients, 50% of whom had a baseline estimated glomerular filtration rate (eGFR) <60 mL/min/1.73 m^2^, the use of postoperative 20% albumin resulted in an increased incidence of cardiac surgery-associated acute kidney injury (CSA-AKI) (48.9% vs 43.4%; adjusted Relative Risk (RR) 1.12, 95% CI 1.04–1.21) and an increased need for blood transfusions [4]. A large retrospective analysis also found that intraoperative 20% albumin increased the risk of CSA-AKI, particularly in patients with normal or elevated preoperative albumin levels [5]. Based on these findings, multiple contemporary guidelines recommend against routine albumin use for CPB priming or perioperative volume replacement in adult cardiac surgery [6, 7, 8]. These trials primarily evaluated short- to medium-term outcomes (up to 90 days) following albumin infusions. More studies with longer follow-up which assess recovery of organ function, hospital re-admissions, and patient-reported quality of life, are still needed to fully determine the clinical value of albumin therapy during cardiac surgery.

Albumin still remains the most physiological colloid and the preferred fluid when a colloid is required, given the well-documented risks of synthetic colloids, which include anaphylaxis, coagulopathy, and AKI [9, 10, 11, 12, 13]. In the era of precision medicine, its role should be considered within a more refined fluid management approach, both in its clinical application and in research, with attention not only to whether albumin should be administered, but also to the appropriate timing, dosing, and concentration. The following sections discuss three patient groups in whom targeted albumin administration could be of potential benefit and may warrant further investigation.

**Severe hypoalbuminemia** (e.g., serum albumin <30 g/L): A low serum albumin is a well-established predictor for adverse outcomes in cardiac surgery [14]. Beyond being a marker of illness or malnutrition, hypoalbuminemia directly contributes to edema, impairs drug binding, and delays healing. It is therefore reasonable to consider albumin supplementation before or during surgery in these patients. The off-pump coronary artery bypass grafting (CABG) trial by Lee *et al*. [15] demonstrated that giving 100–300 mL of 20% albumin (dosed according to baseline albumin level) at the induction of anesthesia reduced the incidence of postoperative AKI. In contrast, a retrospective study in on-pump patients found no renal protection effect of intraoperative albumin administration in patients with preoperative albumin ≤35, 35.1–37.5, and 37.6–40 g/L [5]. These contradictory results may be related to fundamental differences between off-pump and on-pump procedures. During on-pump procedures, hemodilution, systemic inflammation, and albumin loss are more likely to occur due to the use of the CPB circuit. The timing of administration of albumin (preoperative versus intraoperative) may also be critical and warrants further study.

**Situations requiring strict fluid restriction**: Several studies have reported that albumin, compared with crystalloids, can reduce excessive fluid administration and attenuate positive fluid balance [1, 16], which is particularly important in patients with postoperative low cardiac output or right/left ventricular dysfunction, where even modest fluid overload can exacerbate ventricular filling pressures, impair myocardial performance, and precipitate pulmonary or systemic congestion. Albumin results in greater plasma volume expansion per unit volume than crystalloids, and may help to maintain hemodynamic stability under restrictive fluid strategies [17]. Further studies are needed to determine whether integrating albumin into restrictive fluid protocols for these high-risk subgroups can improve critical outcomes such as mortality, length of stay, and readmission rates.

**Patients at risk for endothelial injury**: Animal studies have demonstrated that albumin can mitigate glycocalyx degradation and help preserve endothelial integrity [18, 19]. A clinical observation study in children with sepsis demonstrated that hypoalbuminemia was associated with glycocalyx damage and worse outcomes, while 20% albumin replacement was linked to improved glycocalyx integrity and better outcomes [20]. In contrast, the only interventional study in off-pump CABG found that 5% albumin did not prevent perioperative syndecan-1 elevation compared with crystalloids [21], consistent with findings from an abdominal surgery trial [22]. Potential explanations for these negative findings include patient heterogeneity (most were not hypoalbuminemic), variability in albumin concentration and dosing, and the complex interplay of perioperative factors such as fluid balance. Nevertheless, patients with a high risk of endothelial injury, such as those undergoing prolonged CPB, complex or redo surgery, or experiencing systemic inflammatory states, may still benefit from albumin therapy. Future research should specifically evaluate albumin therapy in these high-risk populations and investigate biomarker-guided strategies. The development of rapid, real-time indicators of glycocalyx injury could ultimately enable individualized and timely albumin administration.

In conclusion, the apparent gap between albumin’s strong physiological rationale and its modest benefits in non-randomized patients highlights the need for a more individualized approach to albumin administration. As discussed in this review, potential beneficial subgroups may include patients with hypoalbuminemia, those requiring strict fluid restriction, and individuals at high risk of endothelial injury. Future research should not only identify patient subgroups most likely to benefit, but also standardize key variables of administration, including weight-based dosing regimens, titration guided by real-time monitoring of oncotic pressure or fluid balance, and adjustments according to baseline renal function and serum albumin levels. Addressing these factors will be critical to developing evidence-based protocols for targeted albumin therapy in perioperative cardiac surgery.

## Availability of Data and Materials

Not applicable.
